# Schizophrenia Polygenic Risk and Brain Structural Changes in Methamphetamine-Associated Psychosis in a South African Population

**DOI:** 10.3389/fgene.2020.01018

**Published:** 2020-10-02

**Authors:** Ruth V. Passchier, Dan J. Stein, Anne Uhlmann, Celia van der Merwe, Shareefa Dalvie

**Affiliations:** ^1^SA MRC Unit on Risk & Resilience in Mental Disorders, Department of Psychiatry and Neuroscience Institute, University of Cape Town, Cape Town, South Africa; ^2^Department of Child and Adolescent Psychiatry and Psychotherapy, TU Dresden, Dresden, Germany; ^3^The Stanley Center for Psychiatric Research, Broad Institute, Cambridge, MA, United States

**Keywords:** polygenic risk, methamphetamine associated psychosis, Africa, brain measures, schizophrenia

## Abstract

**Background:**

The genetic architecture of psychotic disorders is complex, with hundreds of genetic risk loci contributing to a polygenic model of disease. Overlap in the genetics of psychotic disorders and brain measures has been found in European populations, but has not been explored in populations of African ancestry. The aim of this study was to determine whether a relationship exists between a schizophrenia-derived PRS and (i) methamphetamine associated psychosis (MAP), and (ii) brain structural measures, in a South African population.

**Methods:**

The study sample consisted of three participant groups: 31 individuals with MAP, 48 with apsychotic methamphetamine dependence, and 49 healthy controls. Using PRSice, PRS was generated for each of the participants with GWAS summary statistics from the Psychiatric Genomics Consortium Schizophrenia working group (PGC-SCZ2) as the discovery dataset. Regression analyses were performed to determine associations of PRS, with diagnosis, whole brain, and regional gray and white matter measures.

**Results:**

Schizophrenia-derived PRS did not significantly predict MAP diagnosis. After correction for multiple testing, no significant associations were found between PRS and brain measures across all groups.

**Discussion:**

The lack of significant associations here may indicate that the study is underpowered, that brain volumes in MAP are due to factors other than polygenic risk for schizophrenia, or that PRS derived from a largely European discovery set has limited utility in individuals of African ancestry. Larger studies, that include diverse populations, and more nuanced brain measures, may help elucidate the relationship between schizophrenia-PRS, brain structural changes, and psychosis.

**Conclusion:**

This research presents the first PRS study to investigate shared genetic effects across psychotic disorders and brain structural measures in an African population. Ancestrally comparable discovery datasets may be useful for future African genetic research.

## Introduction

Methamphetamine-associated psychosis (MAP) is the development of psychosis during, or soon after, intoxication or withdrawal from methamphetamine (MA). MA substance use disorder is a substantial local and global public health burden (Shin et al., 2017) with up to 40% of those who use MA going on to develop psychosis (Glasner-Edwards and Mooney, 2014). Genetic susceptibility has been recognized as a significant risk factor for the development of psychotic disorders such as MAP and schizophrenia (Chen et al., 2003; Grant et al., 2012). However, the genetic architecture of psychotic disorders is complex, having hundreds of risk loci contributing to the polygenic model of disease (Lvovs et al., 2012).

Polygenic risk scoring (PRS) is a method used to elucidate the polygenic nature of complex disorders by measuring the common variant contribution to the phenotype of interest. PRS has become an established method to determine genetic risk not only within, but also across disorders that share similar phenotypes (International Schizophrenia Consortium, 2009). With the use of PRS, evidence has emerged that there is shared genetic risk across psychotic diagnoses, including schizophrenia and bipolar disorder (International Schizophrenia Consortium, 2009; Hamshere et al., 2011; Tesli et al., 2014; Harrisberger et al., 2016; Vassos et al., 2017; Jonas et al., 2019).

Brain structural measures demonstrate heritability and are altered in psychotic disorders (Keshavan et al., 2007; Grant et al., 2012; Miller and Rockstroh, 2016; Jia and Ck, 2018). More recently, significant associations between schizophrenia-derived PRS and variation in brain structural measures, including global cortical thickness and hippocampal volume have been demonstrated in healthy individuals (Jalbrzikowski et al., 2019; Neilson et al., 2019).

To the knowledge of the authors, there have not been any studies using PRS to investigate shared genetic effects across psychotic disorders and brain structural measures in African populations. The majority of neuropsychiatric research, including PRS studies, has investigated European populations (Duncan et al., 2019). Using genetic and imaging data, the aim of this study was to determine whether a relationship exists between a schizophrenia-derived PRS and (i) MAP diagnosis, and (ii) brain structural measures, in a South African population.

## Materials and Methods

### Study Participants

Data for this study were from the case-control study, *Neural correlates of deficits in affect regulation in methamphetamine dependence with and without a history of psychosis* (Uhlmann, 2015). Ethical approval was obtained from the University of Cape Town Human Research Ethics Committee (684/2017). This study comprised individuals with a diagnosis of MAP (*n* = 31), methamphetamine dependence without psychosis (MD) (*n* = 48), and healthy controls (HC) (*n* = 49). MAP and MD diagnoses were made using the Structured Clinical Interview for DSM-IV Axis I disorders (First et al., 2016). Participants were excluded if they had a history of head trauma, other psychiatric or neurological disease, additional substance dependence (other than nicotine), or a seropositive test for HIV. The participants were of different ancestry, including African, European and mixed ancestry. Participants were matched for age and gender.

### Genotyping and Quality Control

Blood or saliva samples (using the Oragene DNA OG-500 kits) were obtained from each of the participants with the appropriate informed consent. DNA was extracted from blood using the salting out method (Miller et al., 1988) and where saliva samples were collected, DNA was extracted using the manufacturer’s guidelines^1^. Samples were genotyped using the Illumina^®^ Infinium PsychArray at the Broad Institute (Cambridge, MA, United States). Genotyping data was available for 588,454 variants. Using Plink v1.9 (Chang et al., 2015; Purcell and Chang, 2015), the following quality control (QC) steps were performed: removal of duplicate samples, relatedness check where individuals with pi-hat >0.2 were removed, Hardy-Weinberg Equilibrium (HWE) checks (*p* < 0.001), variants with a minor allele frequency (MAF) <0.05 were removed; variants with >10% missing genotype rate were removed.

### Imputation and Post-imputation Quality Control

The Michigan Imputation Server (U.S. National Institutes of Health, 2020) was used to impute the genotype data. For this, the 1000 Genomes (1000G) phase3 v5 ref panel (Auton et al., 2015) was used and the rsq filter was set at 0.3 (estimate of the squared correlation between imputed and true genotypes). Phasing was performed using Eagle v2.4. Post-imputation QC consisted of Hardy-Weinberg Equilibrium (HWE) checks (*p* < 1e-6), variants with a minor allele frequency (MAF) <0.05 were removed; and variants with >10% missing genotype rate were removed. After QC, 128 individuals and 8,249,215 variants remained for downstream analysis.

### Structural Brain Imaging

Structural MRI images were acquired using a 3T Siemens Magnetom Allegra at the Cape Universities Brain Imaging Centre. A radiologist, blinded to diagnosis, examined each scan for structural abnormalities. MRI scans were analyzed using the FreeSurfer software package v5.3^2^ and images were quality controlled following the ENIGMA protocol^3^. Brain measures and regions of interest which have been previously found to be structurally or genetically associated with psychotic disorders, including MAP and schizophrenia, were chosen for analysis: (1) total brain volume; (2) total white matter (WM) volume; (3) cortical thickness; (4) hippocampal volume; (5) inferior temporal gyrus WM volume; and (6) superior temporal gyrus WM volume.

### Principal Component Analysis (PCA)

To account for population stratification, principal components (PC) were calculated from a set of independent SNPs using flashPCA (Abraham and Inouye, 2014), with the 1000G dataset as a reference panel (Auton et al., 2015). The first two PCs were plotted using R (package *ggplot2*) (R Core Team, 2016; Wickham, 2016; Supplementary Figure 1). Prior to calculating the PCs, pairwise LD pruning was conducted with a window size of 1,000 variants and an r^2^ threshold of 0.05 using Plink v2.0 (Chang et al., 2015; Purcell and Chang, 2015).

### PRS

PRS combines the effects of risk variants at specified *p*-value thresholds from a “discovery” GWAS, into a single risk score. The number of alleles an individual in the “target” dataset possesses for a particular variant, is weighted by the effect size of that variant in the “discovery” dataset (International Schizophrenia Consortium, 2009). To assess whether the aggregate scores reflect risk of disease, a mean risk score in target cases is compared to controls (International Schizophrenia Consortium, 2009). The discovery dataset was the Psychiatric Genetic Consortium Schizophrenia (PGC-SCZ2) GWAS summary statistics, comprising 102,636 SNPs^4^ (Ripke et al., 2014; Psychiatric Genomics Consortium, 2016). This large database is made up of 49 ancestry matched, non-overlapping case-control samples (46 of European and three of east Asian ancestry) with 34,241 cases and 45,604 controls; and 3 family based samples of European ancestry (1,235 parent affected-offspring trios). PRS were calculated in the target sample (*n* = 128) using PRSice-2 (Choi and O’reilly, 2019) at multiple *p*-value thresholds (P_T_) (0.001, 0.05, 0.1, 0.2, 0.3, 0.4, 0.5, 1). As a default in PRSice, LD pruning was performed whereby variants were pruned if they were nearby (within 250 kb) and in LD (*r*^2^ > 0.1) with the leading variant (lowest *p*-value) in a given region. Distribution of explained variance (*R*^2^) and optimal *p*-value threshold (*p*_T_) were calculated. To generate the best-fit PRS, MAP diagnosis was used as the primary outcome measure where the first five PCs, age and sex were included as covariates. The proportion of variance explained by PRS was estimated as the difference in Nagelkerke’s R^2^ between the full model (including PRS plus covariates) and the null model (only covariates).

### Statistical Analysis

The Shapiro-Wilk test was used to determine whether the PRS were normally distributed and Levene’s test was used to determine whether there was homogeneity of variance across groups (Shapiro and Wilk, 1965; Schultz, 1985). A *t*-test was performed to test for difference in mean PRS between the MAP and the combined MD and HC groups (apsychotic group). A Welch one-way test was then performed to test for difference in mean PRS between all three groups, MAP, MD, and HC (Welch, 1947). Area under the receiver-operator characteristic curve (AUROC) was calculated to evaluate the ability of a schizophrenia-derived PRS to accurately predict MAP diagnosis in this population.

Linear regression was used to determine the association between best-fit PRS and brain structural measures, with the first five PCs, sex, age, and intracranial volume included as covariates. Participants with missing brain measures were removed. Brain measures were log transformed to improve the model fit and reduce the residual standard error. Significance was set at a Bonferroni-corrected *p*-value threshold of <0.005 (0.05/10 brain measures). Logistic regression was done to test association between MAP diagnosis and brain regional measures. All analyses were performed in the statistical environment, R (R Core Team, 2016).

### *Post-hoc* Power Calculation

The “avengeme” *R* package (Dudbridge, 2013) was used to calculate the sample size necessary to achieve 80% power. With a disease prevalence of 1%, 40,755 overlapping independent SNPs between the discovery and target datasets, and *n* = 128 for the target dataset, a total of 98,885 samples are required in the discovery dataset to attain this level of power. However, to achieve 80% power with the specified target sample size, a p-value threshold of 0.99, for selecting markers into the PRS, is required.

## Results

### Sample Demographics

The demographics of the study sample are listed in Table 1. The majority of the sample was male (*n* = 100) with a mean age of 27 (6) years. The ancestries of the target sample were 97 (75.8%) mixed ancestry, 21 (16.4%) African, and 10 (7.8%) European.

**TABLE 1:** Participant demographics.

Participant group	Number	Age Mean (±SD) (years)	Gender male N (%)	Mixed ancestry N (%)	Black African N (%)	Caucasian N (%)
MAP	31	25 (7)	23 (74.2)	21 (67.7)	9 (29)	1 (3.2)
MD	48	27 (5)	36 (75)	46 (95.8)	2 (4.2)	0
HC	49	28 (7)	39 (79.6)	30 (61.2)	10 (20.4)	9 (18.4)
Total	128	27 (6)	100 (76.6)	97 (75.8)	21 (16.4)	10 (7.8)

**MAP, Methamphetamine Associated Psychosis; MD, Methamphetamine Dependence; HC, Healthy Control; SD, Standard Deviation; N, number.**

### PRS Nagelkerke *R*^2^ and AUC

The best fit PRS at a *P*_T_ = 0.0099 (explaining 4.2% of the variance in MAP, *p* = 0.05) was used for downstream analysis (Supplementary Figure 2). PRS showed normal distribution across groups. There was no significant difference in mean PRS between MAP and AP participant groups [*t* = −1.5086, *df* = 126, *p* = 0.1339, 95% CI (−2.30 × 10^–4^, 3.10 × 10^–5^)]. There was no significant difference in means when groups were further split into MAP, MD and HC [*F*(2, 70) = 2.49, *p* = 0.09]. Using the PRS as the predictor, and MAP participant group as the outcome, the corrected partial area under the curve was 53.4% (95% CI: 49.7–61.6%).

### Brain Measures

A total of 18 samples were removed due to missing brain measures, leaving 27 MAP patients and 83 AP patients in the regression analyses. After correction for multiple testing, there were no statistically significant association between PRS and any of the log transformed brain measures across all groups. These results are summarized in Table 2. The strongest associations were observed in left inferior temporal WM volume (β = −9.82 × 10^2^, *p* = 0.02) and left superior temporal WM volume (β = −1.12 × 10^3^, *p* = 0.009). No significant associations were found between any of the brain measures and MAP diagnosis. The differences in brain measures between groups was not the focus of this research, but has been published elsewhere (Uhlmann et al., 2016).

**TABLE 2:** Linear regression results for brain structural measures and polygenic risk score.

Brain measure^a^	β	*t*-value	*P*-value	(Adjusted) *R*^2^
Left hippocampal volume	2.857×10^2^	0.602	0.549	0.016
Right hippocampal volume	−4.059×10^2^	–0.957	0.341	–0.002
Right inferior temporal white matter volume	−6.691×10^2^	–1.502	0.136	0.023
Left superior temporal white matter volume	−1.219×10^3^	–2.680	0.009	0.015
Right superior temporal white matter volume	−2.821×10^2^	–0.667	0.506	–0.010
Total brain volume	–61.002244	–0.899	0.371	0.305
Right hemisphere cortical thickness	5.251×10^2^	–1.055	0.294	–0.002
Left hemisphere cortical thickness	−5.484×10^2^	–1.186	0.239	0.029
Total white matter volume	3.648×10^2^	0.825	0.412	0.067
Left inferior temporal white matter volume	−9.816×10^2^	–2.358	0.020	0.055

*^*a*^Covariates included age, sex, intracranial volume for all measures.*

## Discussion

In this study, we sought to determine whether PRS derived from a well-powered schizophrenia GWAS, comprising mainly European populations, was able to predict MAP and brain volume and thickness in an ancestrally diverse South African target sample. After correction for multiple testing, we did not identify a significant association between schizophrenia-derived PRS and MAP diagnosis, or any of the brain structural measures. This is in contrast to a study conducted in an Asian population group which showed that a large number of “risk” alleles for MAP were over-represented in individuals with schizophrenia. However, it is worth noting that the overlap from this previous study was only able to explain 0.7% of the variance in schizophrenia liability (Ikeda et al., 2013).

There are a number of different explanations for the null findings obtained here. In particular, polygenic risk scores for schizophrenia may not be associated with measures of brain volume in healthy individuals or in those with psychotic disorders. This explanation is consistent with findings from previous research in populations of largely European ancestry (Reus et al., 2017; Harrisberger et al., 2018; Lancaster et al., 2018; Simões et al., 2020). For example, no associations between PRS for schizophrenia and for bipolar disorder with either subcortical volume or WM microstructure, were found in the United Kingdom Biobank (Reus et al., 2017). Similarly, in healthy subjects with higher genetic risk for schizophrenia, based on loci found to be associated with schizophrenia (Psychiatric Genomics Consortium, 2016), no consistent associated brain volume changes were observed (Van Der Auwera et al., 2017). Furthermore, a systematic review established that schizophrenia-derived PRSs were not significantly associated with brain structural changes in five out of the seven studies included (Van Der Merwe et al., 2018). As the knowledge and evidence of imaging genetics increases, more nuanced structural and functional brain measures are being studied. For example, measures of brain connectivity and of task-dependent recruitment of multiple brain regions may be associated with genetic variation in psychosis (Ranlund et al., 2017; Dezhina et al., 2019; Cao et al., 2020). Added to this, advanced methodological approaches integrating environmental exposures, gene-gene interactions, and epigenetics from a variety of ancestral cohorts, are needed to fully appreciate missing heritability (Mufford et al., 2017; Alnæs et al., 2019).

Caution is also needed when interpreting studies with differing ancestries in the discovery and target datasets. Correlations between true (discovery population) and inferred (target population) risk are highest in the population from which summary statistics are derived (Martin et al., 2017; Mostafavi et al., 2019). Therefore, scores are fundamentally less informative in populations more diverged from the discovery GWAS study cohorts (Scutari et al., 2016; Martin et al., 2019). The majority of neuropsychiatric genetic studies have been undertaken in high-income settings, thus GWAS summary statistics are limited to mostly individuals of European and, more recently, Asian ancestry (Martin et al., 2017; Duncan et al., 2019). A review of PRS performance in diverse human populations revealed that, with current available genetic discovery datasets, people of African descent have the lowest polygenic score performance, compared to all other populations tested, including Latino, Middle Eastern, and East and South Asian. Indeed, predictive performance of European ancestry-derived PRS in populations of African descent is only 42% of that of matched European ancestry samples- almost halving the potential of studies such as our own (Martin et al., 2017; Vassos et al., 2017; Duncan et al., 2019).

This points to what is needed next: targeted large-scale genetic investigation of schizophrenia and other psychiatric disorders in African populations. Such research might ensure that future developments, and the clinical utility, of PRS will be equally applicable to health care users of African descent and limit exacerbating already existing health disparities (Martin et al., 2019). Projects under way, such as the Neuropsychiatric Genetics of African Populations-Psychosis (NeuroGAP-Psychosis), are expanding knowledge of the genetic and environmental risk architecture of neuropsychiatric disorders in African populations of South Africa, Ethiopia, Kenya and Uganda (Stevenson et al., 2019). This will improve the availability of ancestrally comparable discovery datasets and meaningful results for African genetic research. In further support of this call to action, it has been recognized that African genomes contain more diversity than any other population group, having uniquely shorter haplotype blocks with more variants per individual (Genomes Project Consortium, 2015). As a result, studies of African populations could uncover additional pathogenic variants and identify novel disease-associated loci (Dalvie et al., 2015). Therefore, future African neuropsychiatric research may yield genomic insights into the risk, resilience (Wojcik et al., 2019), and treatment of psychiatric disorders, advancing precision medicine across global populations (Dalvie et al., 2015).

This study has several limitations that deserve emphasis. Firstly, the target group was relatively small, and comprised participants of different ancestral groups. Although the use of PCAs as covariates in the PRS is a robust method for correcting for population stratification (Wu et al., 2011), it cannot resolve all bias due to differences in the population sample (Price et al., 2006). Ideally, the study sample should be stratified into ancestrally homogenous groups and analyzed separately (Duncan et al., 2019). Secondly, psychiatric diagnosis was made on the basis of a single diagnostic interview, which may lead to misclassification (Schijven et al., 2020). Future research on MAP may benefit from longitudinal study designs which ensure longitudinal expert assessment and diagnosis. Lastly, the brain structural changes observed in previous psychiatric research have been subtle, are highly heterogeneous, and are influenced by multiple potential confounding factors (Brent et al., 2013; Weinberger and Radulescu, 2015). Such confounding effects include: demographics, frequency of MA use, long term neuroleptic treatment, participant movement, hydration and stress during scan, which were not accounted for in this study (Yudofsky and Hales, 2004; Streitbürger et al., 2012; Weinberger and Radulescu, 2015; Mufford et al., 2017; Arunogiri et al., 2018).

## Conclusion

This research is the first to use PRS to investigate shared genetic effects across psychotic disorders and brain structural measures in an African population. Ancestrally comparable datasets and more nuanced structural and functional brain measures may be useful in further elucidating the genetic risks for psychotic disorders including MAP.

## Data Availability Statement

The datasets generated for this article are not publicly available The primary data was collected using a patient consent form that did not include consent to deposit genotyped data into a public repository. Requests to access the datasets should be directed to AU, uhlmann.aa@gmail.com.

## Ethics Statement

This study was reviewed and approved by the University of Cape Town Human Research Ethics Committee (approved 684/2017). The participants provided written informed consent.

## Author Contributions

All authors contributed to the article and approved the submitted version. RP: study design, statistical and genetic analysis, draft of manuscript, and revisions. DS: study design, review of manuscript, and editing. AU: collection of primary data and review of manuscript. CM: review of manuscript. SD: study design, statistical and genetic analysis, review and revisions of manuscript.

## Conflict of Interest

The authors declare that the research was conducted in the absence of any commercial or financial relationships that could be construed as a potential conflict of interest.

## Footnotes

1 https://www.dnagenotek.com/us/pdf/PD-PR-006.pdf

2 http://surfer.nmr.mgh.harvard.edu/

3 http://enigma.ini.usc.edu/protocols/imaging-protocols

4 http://pgc.unc.edu
