# Supplementary figures and images for: Schizophrenia Polygenic Risk and Brain Structural Changes in Methamphetamine-Associated Psychosis in a South African Population
Source: Front Genet. 2020 Oct 2;11:1018. doi: 10.3389/fgene.2020.01018 (PMC7566162; doi:10.3389/fgene.2020.01018)

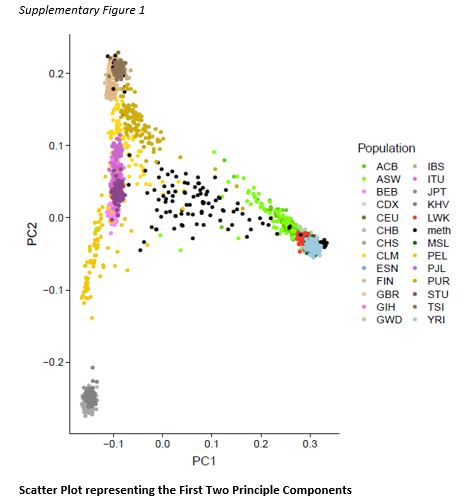

Supplement: Supplementary file 1 [file Image_1.jpg]

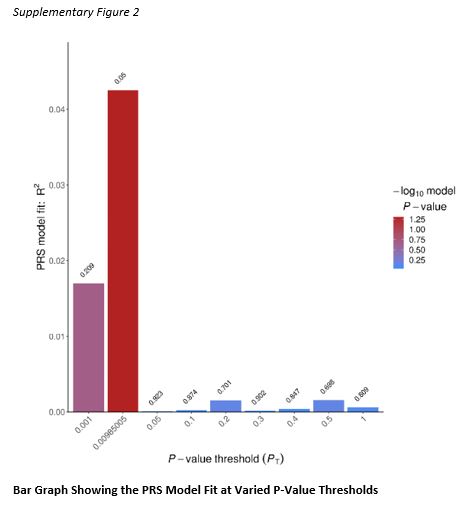

Supplement: Supplementary file 2 [file Image_2.jpg]
